# Synergic Activation of Toll-Like Receptor (TLR) 2/6 and 9 in Response to *Ureaplasma parvum* & *urealyticum* in Human Amniotic Epithelial Cells

**DOI:** 10.1371/journal.pone.0061199

**Published:** 2013-04-12

**Authors:** Martha Triantafilou, Benjamin De Glanville, Ali F. Aboklaish, O. Brad Spiller, Sailesh Kotecha, Kathy Triantafilou

**Affiliations:** Department of Child Health, School of Medicine, University Hospital of Wales, Cardiff University, Heath Park, Cardiff, United Kingdom; Institute of Microbial Technology, India

## Abstract

*Ureaplasma* species are the most frequently isolated microorganisms inside the amniotic cavity and have been associated with spontaneous abortion, chorioamnionitis, premature rupture of the membranes (PROM), preterm labour (PL) pneumonia in neonates and bronchopulmonary dysplasia in neonates. The mechanisms by which *Ureaplasmas* cause such diseases remain unclear, but it is believed that inappropriate induction of inflammatory responses is involved, triggered by the innate immune system. As part of its mechanism of activation, the innate immune system employs germ-lined encoded receptors, called pattern recognition receptors (PRRs) in order to “sense” pathogens. One such family of PRRs are the Toll like receptor family (TLR). In the current study we aimed to elucidate the role of TLRs in *Ureaplasma*-induced inflammation in human amniotic epithelial cells. Using silencing, as well as human embryonic kidney (HEK) transfected cell lines, we demonstrate that TLR2, TLR6 and TLR9 are involved in the inflammatory responses against *Ureaplasma parvum* and *urealyticum* serovars. *Ureaplasma* lipoproteins, such as Multiple Banded antigen (MBA), trigger responses via TLR2/TLR6, whereas the whole bacterium is required for TLR9 activation. No major differences were observed between the different serovars. Cell activation by *Ureaplasma parvum* and *urealyticum* seem to require lipid raft function and formation of heterotypic receptor complexes comprising of TLR2 and TLR6 on the cell surface and TLR9 intracellularly.

## Introduction

Intrauterine infection is a major cause of spontaneous preterm birth (PTB). The association of PTB and colonisation of the cervicovaginal tract with specific microorganisms, especially before 32 wk of pregnancy, has long been recognised.

There is accumulating epidemiologic and experimental evidence that intrauterine or postnatal infection with *Ureaplasma* species is a significant risk factor for adverse pregnancy outcomes and complications of extreme preterm birth such as bronchopulmonary dysplasia and intraventricular hemorrhage [1]. *Ureaplasma* are wall-less bacteria belonging to the *Mycoplasmataceae*. Intraamniotic infection with *Ureaplasma* species has serious complications both for mothers as well as neonates. For the mother, *Ureaplasma* infection is a major risk factor for spontaneous premature rupture of membranes (PROM), clinical chorioamnionitis and preterm delivery [2]; [3]. At least 40% of all preterm births are thought to occur in mothers with *Ureaplasma* infection that is usually chronic, subclinical and asymptomatic up to the time labour begins or the membranes rupture. For the fetus or neonate, infections with *Ureaplasma* are associated with a number of adverse outcomes, including chronic lung disease, pneumonias, cerebral white matter lesions, cerebral palsy and even death [4]; [5]. The mechanisms by which *Ureaplasmas* cause such diseases remain unclear, but it is believed that inappropriate induction of inflammatory responses is involved, most likely triggered by the innate immune system [6]; [7]. It is believed that the innate immune response to these bacteria can lead to the activation of pattern recognition receptors (PRRs), production and release of pro-inflammatory mediators leading to the complications associated with preterm and term infants. Increased levels of pro-inflammatory mediators such as interleukin-6 (IL-6), tumour-necrosis-factor (TNF-α), IL-1βand IL-8 have been shown in amniotic fluid infected with *Ureaplasma*, supporting this hypothesis [8].

As part of its mechanism of activation, the innate immune system employs germ-lined encoded receptors, called pattern recognition receptors (PRRs) in order to “sense” pathogens. One such family of PRRs are the Toll like receptor family (TLR), which has been implicated in bacterial recognition.

The question that remains is which PRRs sense these bacteria, especially since they are wall-less. Very little is known about the innate immune recognition of *Ureaplasma*. There is a single study that implicates TLR1, TLR2 and TLR6 in *Ureaplasma parvum* innate-immune recognition [9], but this study was performed with *U. parvum* serovar 3 (SV3), which was cultured in broths enriched with yeast extract, thus results obtained for the activation of certain TLRs might be attributed to the contaminating yeast components.

In this study we aimed to elucidate the role of TLRs in *Ureaplasma*-induced inflammation. Previously we have shown that *U. parvum* serovar 3 (SV3) was highly sensitive to bactericidal effects of non-immune human serum, while SV14 showed bactericidal resistance to serum from human serum containing high titres of anti-*Ureaplasma* antibodies [10]. Therefore, in this study we set out to compare the relative stimulation of TLRs by these two isolates, which are closely related, yet separate species of *Ureaplasma*.

## Materials and Methods

### Cells

HEK293 cells transfected with either TLR2, TLR2/TLR6 or TLR4 were kindly provided by Professor Douglas Golenbock (University of Massachusetts Medical School, Worcester, USA). HEK293 cells transfected with either TLR7 or TLR9 were obtained from Invivogen (USA).

Transfected cell lines were maintained in Dulbecco's modified Eagle's medium supplemented with 10% fetal calf serum, 0.5 units/ml penicillin, 0.5 µg/ml streptomycin, 400 µg/ml G418 and 10 µg/ml Ciprofloxacin for HEK/TLR2, HEK/TLR2/TLR6, and HEK/TLR4 and 10 µg/ml blasticidin for HEK/TLR7, and HEK/TLR9.

Primary human amniotic epithelial cells were purchased from TCS Cells Works (Buckingham, UK). The cells are isolated according to referenced procedures. Human amniotic epithelial cells are isolated from human amniotic membranes. Each isolate undergoes extensive testing for the presence of specific amniotic epithelial cell markers.

### Ureaplasma parvum & urealyticum

*Ureaplasma parvum* SV3 (HPA5) and SV14 (HPA32) are clinical isolates that have previously been characterised for susceptibility to bactericidal serum activity [10], as well as *Ureaplasma urealyticum* SV2 (Cook strain) which was obtained from the American Type culture collection (ATCC). *Ureaplasma* were sub-cultured in Ureaplasma selective medium obtained from Mycoplasma Experience (Surrey, U.K.). Strains were propagated in complete medium as well as medium lacking yeast and all data were compared to determine if propagation in the presence of yeast altered the results. *Ureaplasma* yields were not adversely affected by the absence of yeast in the culture medium and data presented exclude any confounding effects of yeast proteins.

20 ml of bacteria were harvested by centrifugation 12,000 rpm for 20 min. The pellet was washed three times in PBS and resuspended in 500 µl of PBS. The cell number was determined by absorbance (A_600_×0.1 = 10^8^ bacteria/ml. For stimulation we used 1×10^8^ bacteria/ml to 1×10^7^ cells/ml.

### Materials

All chemicals were obtained from Sigma (Dorset, UK). TLR1, TLR2, TLR4, TLR6, TLR7, and TLR9 specific polyclonal antibodies were obtained from Santa Cruz Biotechnology Inc. (Heidelberg, Germany). Recombinant Multiple Banded Antigen of Ureaplasma (MBA) was obtained from Genway Biotech Inc (San Diego, USA). The recombinant MBA was passed through a Profos Endotrap® blue 10 column obtained from Hyglos (Munich, Germany) in order to ensure there was no LPS. Different concentrations were tested for IL-6 secretion and optimum results were obtained with 1 µg/ml, thus this concentration was used for all subsequent activation assays.

### Flow cytometric determination of TLR expression

In order to investigate TLR expression before and after *Ureaplasma* infection, human amniotic epithelial cells were either stimulated with *Ureaplasma* serovars for 1 h or not, prior to fixation with 4% paraformaldehyde. The cells were subsequently washed and permeabilised using PBS/0.02%BSA/0.02% Saponin. After permeabilisation, the cells were incubated with antibodies against different TLRs and the appropriate secondary antibodies conjugated to FITC. The cells were washed twice in PBS/0.02% BSA/0.02% Saponin and resuspended in 500 µl of PBS. Fluorescence was detected using a FACSCalibur counting 10,000 cells not gated.

### Cytokine assays

Human amniotic epithelial cells were either not stimulated or stimulated with *Ureaplasma* SV3, SV14 or SV2 (1×10^8^ bacteria/ml to 1×10^7^ cells/ml) or MBA (1 µg/ml). The cultures were incubated for the designated times. The supernatants were collected and frozen until the cytokine assays were performed. The BectonDickinson Human Inflammation cytometric bead array system (Oxford, UK) was used in order to determine the level of multiple cytokines at the same time.

### RNA interference

RNA interference was used in order to silence the TLR2, TLR4, TLR6, TLR7 and TLR9 genes. psiRNA clones were obtained from InvivoGen (San Diego, USA). Human primary amniotic epithelial cells (1×10^5^) were seeded in six well plates and transfected with 0.5 µg of psiRNA using Lipofectamine 2000 (Invitrogen, Paisley, UK). After 48 h the level of silencing was determined and cells were used for activation assays.

### Cell labelling for FRET

Human amniotic epithelial cells were labelled with 100 µl of a mixture of donor conjugated antibody Cy3 and acceptor conjugated antibody Cy5. The cells were either not stimulated, or stimulated with Ureaplasma parvum, urealyticum or MBA for 1 h, and were rinsed twice in PBS/0.02% BSA, prior to fixation with 4% formaldehyde for 15 min. The cells were fixed in order to prevent potential re-organisation of the proteins during the course of the experiment.

Cells were imaged on a Carl Zeiss, Inc. LSM510 META confocal microscope (with an Axiovert 200 fluorescent microscope) using a 1.4 NA 63× Zeiss objective. The images were analysed using LSM 2.5 image analysis software (Carl Zeiss, Inc.). Cy3 and Cy5 were detected using the appropriate filter sets. Using typical exposure times for image acquisition (less than 5 s), no fluorescence was observed from a Cy3-labelled specimen using the Cy5 filters, nor was Cy5 fluorescence detected using the Cy3 filter sets.

### FRET measurements

FRET is a non-invasive imaging technique that can be used in order to study molecular associations. It involves non-radiative transfer of energy from the excited state of a donor molecule to an appropriate acceptor. The rate of energy transfer is inversely proportional to the sixth power of the distance, between donor and acceptor. In the present study, FRET was measured using a method as previously described.[11], [12], [13].

### Confocal microscopy

Human amniotic epithelial cells on microchamber culture slides (Lab-tek, Gibco), were stimulated with Ureaplasma SV3 or MBA (1 µg/ml) for different time points, and were subsequently rinsed twice in PBS/0.02% BSA, prior to fixation with 4% formaldehyde for 15 min. The cells were fixed in order to prevent potential re-organisation of the proteins during the course of the experiment. Cells were permeabilised using PBS/0.02% BSA/0.02% Saponin and labelled with anti-TLR9, or MyD88 antibody followed by incubation with the appropriate fluorescently-labelled secondary antibody. Cells were imaged on a Carl Zeiss, Inc. LSM510 META confocal microscope (with an Axiovert 200 fluorescent microscope) using a 1.4 NA 63× Zeiss objective.

## Results

### U. parvum SV3, SV14 and U. urealyticum SV2 activation of human amniotic epithelial cells

Human amniotic epithelial cells represent the first line of defense against intra-amniotic infection and thus provide a cell barrier that should be able to recognise and respond the bacteria, such as *Ureaplasma*. A probable result of their embryonic origin, they have been shown to lack major histocompatibility complex antigens but it has recently been shown that they express functional TLRs [14]. Thus initially we investigated whether *Ureaplasma* serovars could induce the production of pro-inflammatory cytokines from human amniotic epithelial cells.

**Figure 1:**
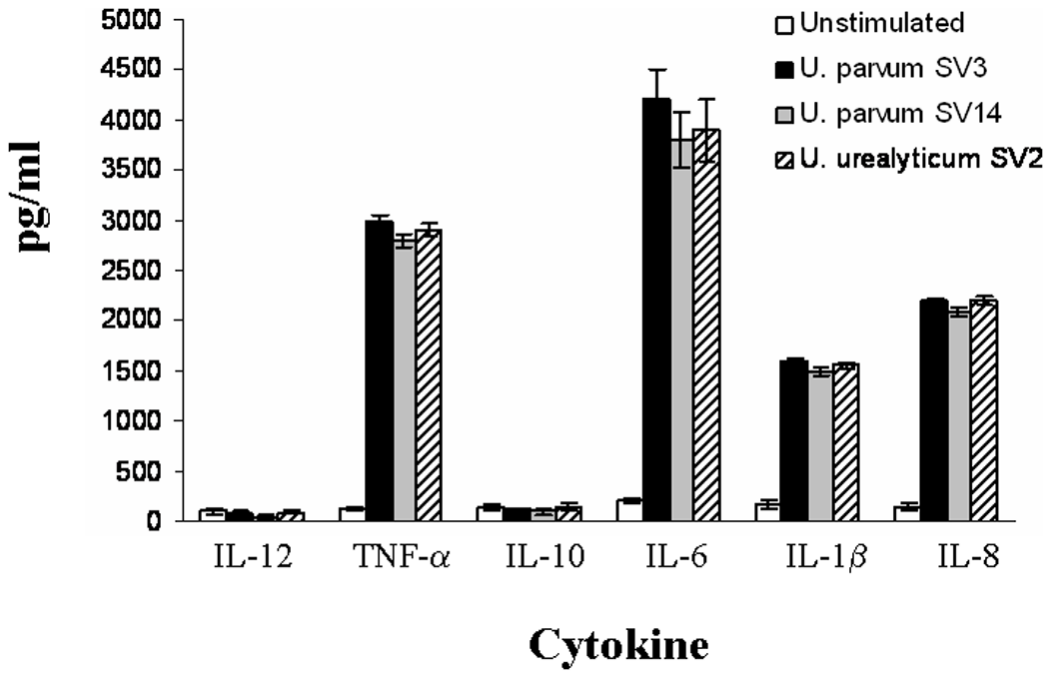
Ureaplasma activation of human amniotic epithelial cells. Human amniotic epithelial cells were incubated with *Ureaplasma* (1×10^8^ bacteria/ml to 1×10^7^ cells/ml) *parvum SV3* (black barcharts), *U. parvum* SV14 (white barcharts) or *U. urealyticum* SV2 (grey bar charts) for 2 h. The supernatants were harvested and assayed for cytokine contents using the Cytometric Bead Array (CBA) system (Becton Dickinson). Fluorescence was detected using a FACSCalibur (BectonDickinson). The data represents the mean ± SD of three independent experiments.

Human amniotic epithelial cells were incubated with *Ureaplasma* SV2, SV3, and SV14 for different time points and the supernatants were assayed for inflammatory cytokines. It was shown that *Ureaplasma* serovars can induce TNF-α, IL-6, IL-1β and IL-8 in human amniotic epithelial cells (Figure 1). Innate immune reponses constitute the first line of host defence, thus pro-inflammatory cytokines were found to be triggered rather quickly and peaked within the first two hours. Therefore, it was decided to stimulate the cells for 2 h for all subsequent stimulations.

### TLR expression on human amniotic epithelial cells

Several TLRs have been implicated in the innate recognition of bacteria. Since *Ureaplasma* are wall-less bacteria it is very interesting to identify which TLRs are able to sense it, thus we investigated the total TLR expression on human amniotic epithelial cells before and after stimulation by *Ureaplasma*.

**Figure 2:**
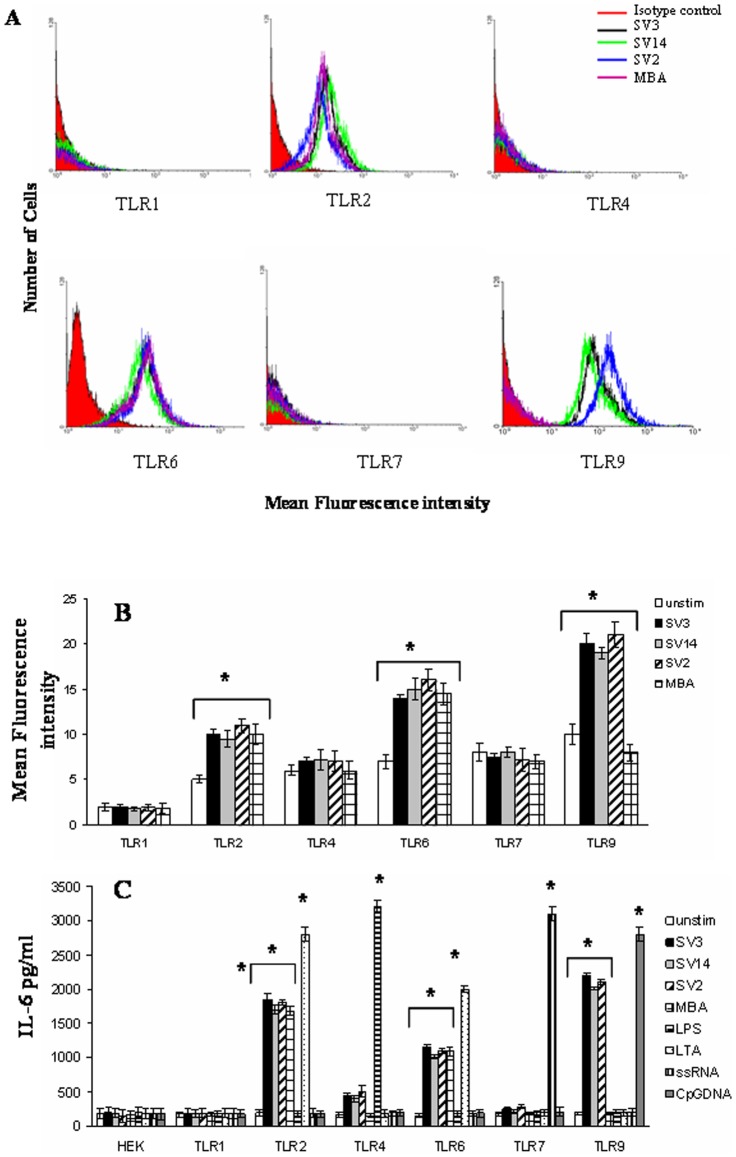
TLR2/6 and TLR9-dependent cytokine secretion in response to Ureaplasma serovars. Human amniotic epithelial cells (A, B) were either not stimulated (white bar charts) or stimulated with Ureaplasma serovars for 2 hours. The cells were fixed and permeabilised, followed by antibody staining against the particular TLR molecule, and incubation with the appropriate secondary antibody conjugated to FITC. Fluorescence was detected using a FACSCalibur (BectonDickinson The data presented is the mean of three independent experiments. HEK-293 cells (B) transfected with TLR1, TLR2, TLR2/6, TLR4, TLR7 and TLR9 were either not incubated (white bar charts) or incubated with with *Ureaplasma* (1×10^8^ bacteria/ml to 1×10^7^ cells/ml) *parvum SV3* (black barcharts), *U. parvum* SV14 (grey barcharts), *U. urealyticum* SV2 (stripped bar charts), or MBA (1 µg/ml) for 2 h. Control cultures were stimulated with known TLR2, TLR4, TLR7 or TLR9 ligands. The supernatants were harvested and assayed for IL-6 content using the Cytometric Bead Array (CBA) system (Becton Dickinson). Fluorescence was detected using a FACSCalibur (BectonDickinson). The data represents the mean ± SD of three independent experiments. Asterisks indicate statistically significant (*p*<0.05) increase in expression (A,B) or IL-6 secretion (C) compared to corresponding unstimulated controls.

We found that human amniotic epithelial cells expressed minimal levels of all TLRs that we tested (TLR1-9) prior to *Ureaplasma* stimulation (Figure 2A). Interestingly, it was shown that the expression of TLR2, TLR6 and most significantly TLR9 increased upon stimulation with *Ureaplasma* serovars (Figure 2A).

In order to investigate whether the effects observed are due to the recognition of lipoproteins from *Ureaplasma* as it has previously been suggested [9], we proceeded to stimulate human amniotic epithelial cells with Multiple Banded Antigen (MBA). The MBA is a surface exposed lipoprotein, which can undergo size and phase variation *in vitro* and *in vivo* [15]; [16]. The MBA is predicted to be a major ureaplasmal virulence factor and is the predominant antigen recognised by sera during infections in humans [17]. MBA was found to increase the expression of TLR2 and TLR6, but not TLR9 in human amniotic epithelial cells (Figure 2A).

### Ureaplasma-induced activation is mainly TLR2/6–dependent on the cell surface

In order to investigate which TLRs might play a role in *Ureaplasma*-induced activation we utilised transfected cell lines. Human embryonic kidney (HEK) cells transfected with either TLR1, TLR2, TLR2/TLR6, TLR4/MD2, TLR7 or TLR9 were utilised. Untransfected HEK cells, which do not express TLRs, did not produce IL-6 in response to *Ureaplasma* species (Figure 2B). Similarly *Ureaplasma* did not trigger cytokine production in HEK cells transfected with TLR7. HEK-TLR4 cells were shown to produce a very small amount of IL-6 in response to *Ureaplasma* serovars, but not in response to MBA (Figure 2B), suggesting that TLR4 does not recognise MBA, but rather some other antigen. On the contrary, HEK cells transfected with either TLR2, TLR2/6 or TLR9 produced significant levels of IL-6 after incubation with *Ureaplasma* serovars. MBA was able to trigger responses only in TLR2 and TLR2/6 expressing cells, suggesting that TLR2/6 heterodimers must recognise these lipoproteins on the cell surface. Interestingly HEK cells transfected with TLR9 had the highest IL-6 response against *Ureaplasma* species (Figure 2B), thus suggesting that *Ureaplasma*-induced activation is mediated mainly through TLR2/6 on the cell surface and TLR9 intracellularly. Control cultures were stimulated with known TLR2, TLR4, TLR7 or TLR9 ligands. It was shown that *Ureaplasma* species were able to stimulate much weaker immune responses compared to other TLR ligands, such as LPS, LTA, ssRNA or CpGDNA.

### Inhibition of Ureaplasma-induced activation of human amniotic epithelial cells by silencing TLR2, TLR6 and TLR9 and to a lesser extent TLR4

**Figure 3:**
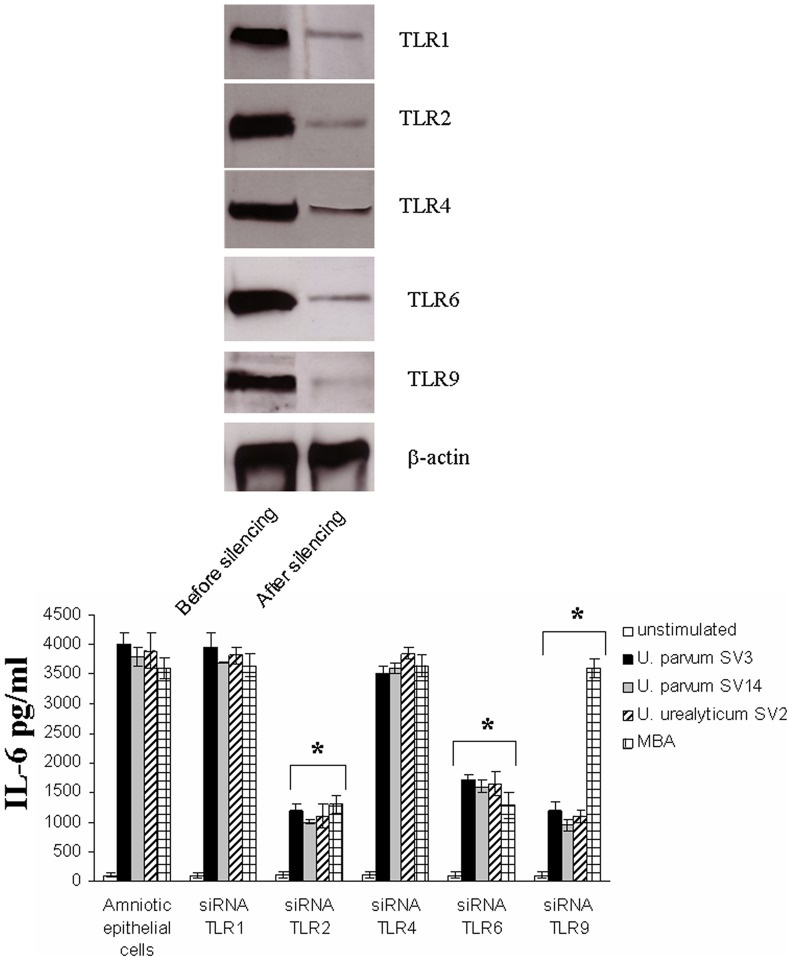
Inhibition of *Ureaplasma* activation of human amniotic epithelial cells by silencing TLR2 and TLR9. TLR expression was knocked down by siRNA and confirmed by western blotting (A). Following RNA interference, human amniotic epithelial cells were not stimulated (white bar chasrts), or stimulated with *Ureaplasma* (1×10^8^ bacteria/ml to 1×10^7^ cells/ml) *parvum SV3* (black barcharts), *U. parvum* SV14 (grey barcharts), *U. urealyticum* SV2 (stripped bar charts) or MBA (1 µg/ml) for 2 h (B). The supernatants were harvested and assayed for cytokine secretion using the Cytometric Bead Array (CBA) system (Becton Dickinson). Fluorescence was detected using a FACSCalibur (BectonDickinson). The data represents the mean ± SD of three independent experiments. Asterisks indicate statistically significant (*p*<0.05) decrease in IL-6 secretion compared to corresponding unsilenced controls.

In order to determine the role of TLR2/6 and TLR9 in *Ureaplasma* recognition, we used RNA interference (siRNA) to knock down the expression of TLR1, TLR2, TLR4, TLR6 and TLR9 in primary human amniotic epithelial cells. Transfection with synthetic TLR specific psiRNA resulted in approximately 70% decrease in TLR1, TLR2, TLR4, TLR6 and TLR9 expression as determined by western blotting (Figure 3A). Control transfections of human amniotic epithelial cells with the psiRNA vector did not affect TLR expression.

Following RNA interference, human amniotic epithelial cells were incubated with *Ureaplasma* SV3, SV14, SV2 as well as MBA (Figure 3B). Cytokine assays were performed after the designated incubation times. It was shown that silencing of TLR2 inhibited *Ureaplasma*- and MBA-induced cellular activation (Figure 3B), thus suggesting the importance of TLR2 in *Ureaplasma*-mediated activation of human amniotic epithelial cells. Although silencing of TLR6 also inhibited *Ureaplasma*-and MBA-induced cellular activation, it was to a lesser extent, thus suggesting that innate immune responses against *Ureaplasma* on the cell surface are triggered mainly through TLR2. Interestingly, silencing of TLR9 did not inhibit MBA-induced activation, but only *Ureaplasma*-induced cytokine response, to a level similar to that of TLR2, thus suggesting that TLR9 is one of the main sensor for *Ureaplasma* intracellularly.

### Ureaplasma-induced receptor clusters on the cell surface amniotic epithelial cells

**Figure 4:**
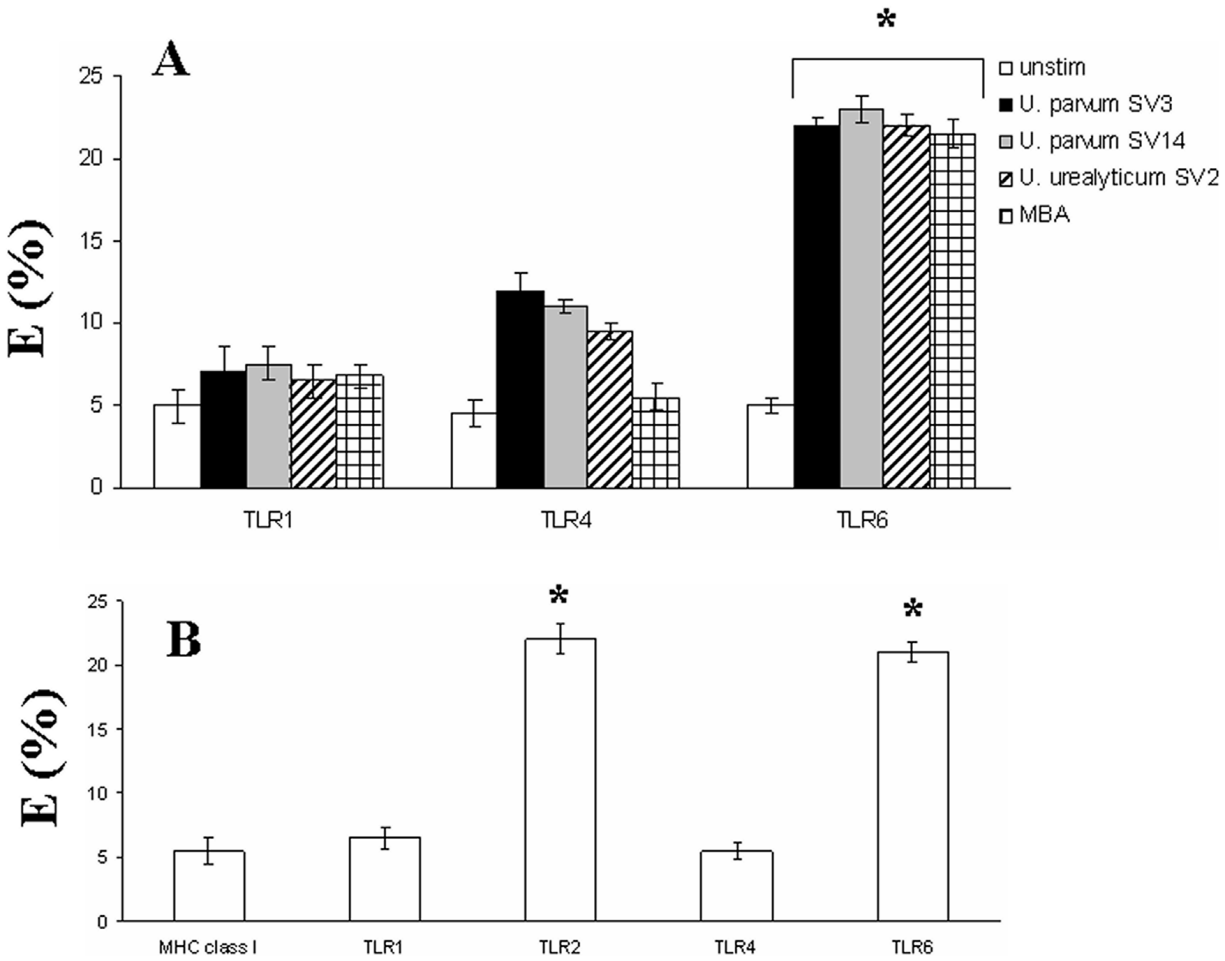
TLR2 heterotypic associations in response to Ureaplasma. (A) Human amniotic epithelial cells were stimulated with no stimulus (white bar charts), or incubated with *Ureaplasma* (1×10^8^ bacteria/ml to 1×10^7^ cells/ml) *parvum SV3* (black barcharts), *U. parvum* SV14 (grey barcharts), *U. urealyticum* SV2 (stripped bar charts) or MBA (1 µg/ml) for 1 h. Energy transfer between TLR2 (Cy3) and the different receptors was measured from the increase in donor (Cy3) fluorescence after acceptor (Cy5) photobleaching. (B) Energy transfer between MBA (Cy3) and the different receptors was measured from the increase in donor (Cy3) fluorescence after acceptor (Cy5) photobleaching. The percentage of energy transfer and standard deviation was calculated from three independent experiments. Asterisks indicate statistically significant (*p*<0.05) increase in energy transfer compared to corresponding unstimulated controls.

In order to investigate whether *Ureaplasma* serovars could induce the formation of receptor activation clusters on human amniotic epithelial cells, we measured FRET in terms of dequenching of donor fluorescence after complete photobleaching of the acceptor fluorophore. We tested the energy transfer efficiency in our system using a positive control, i.e. energy transfer between mAbs to different epitopes on TLR4 molecules, showing that the maximum energy transfer efficiency (E%) was 37 ± 1.2. We proceeded to measure FRET between TLR2 and different PRRs that we had found to be implicated in *Ureaplasma* induced activation, TLR1, TLR6, TLR4. Thus we measured FRET between TLR2 and these molecules in response to the different *Ureaplasma* serovars as well as MBA. TLR2 was found not to associate with these receptor molecules prior to *Ureaplasma* stimulation (Figure 4A, white bars).

Energy transfer between TLR2-Cy3 and the various Cy5-labelled PRRs was also measured. TLR2 was found not to associate with TLR1, but to associate with TLR6, and to a much lesser extent with TLR4 after *Ureaplasma serovar* stimulation. Interestingly when cells were stimulated with MBA, there was no association between TLR2 and TLR4.

In order to determine whether MBA directly interacts with TLR2/6, we measured FRET between MBA-Cy3 and the various Cy5-labelled PRRs (Figure 4B). It was shown that there was energy transfer between MBA-Cy3 and TLR2 as well as TLR6, demonstrating that TLR2/6 heterodimers bind MBA and trigger activation.

Control experiments using the method described by Kenworthy et al.[12] ruled out the possibility that the FRET observed was due to randomly distributed molecules (data not shown).

### Recruitment of TLR2 in lipid rafts following Ureaplasma stimulation on amniotic epithelial cells

**Figure 5:**
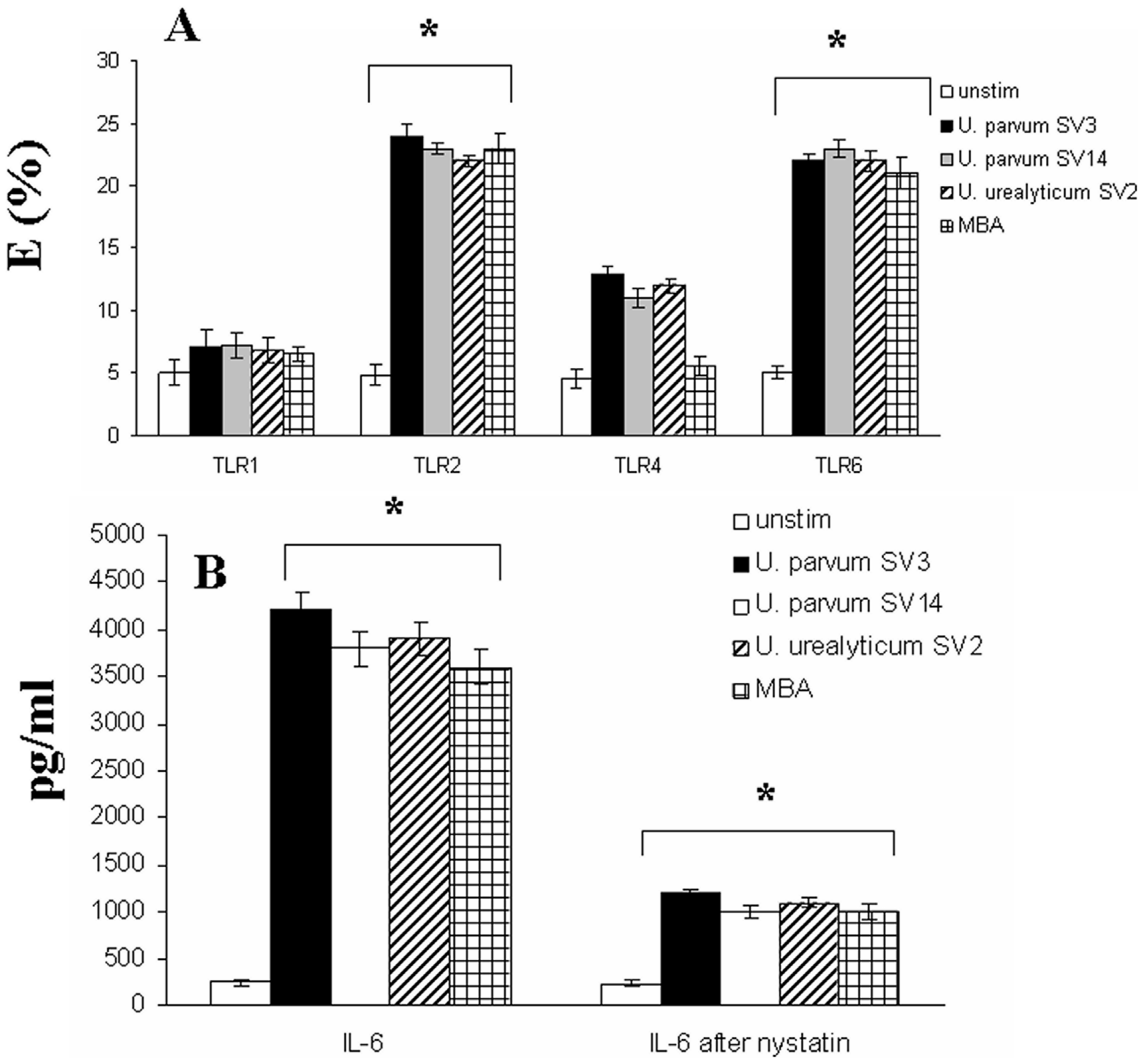
TLR and GM-1 ganglioside FRET mearurements before and after Ureaplasma stimulation. TLR and GM-1 ganglioside FRET mearurements before and after Ureaplasma stimulation of human amniotic epithelial cells (A). Energy transfer between Cy3-labelled TLR1, TLR2, TLR4 or TLR6 and GM-1 ganglioside (Cy5-cholera-toxin) before (white bar charts) and after stimulation with *Ureaplasma* (1×10^8^ bacteria/ml to 1×10^7^ cells/ml) *parvum SV3* (black barcharts), *U.parvum* SV14 (grey barcharts), *U. urealyticum* SV2 (stripped bar charts) or MBA (1 µg/ml). Energy transfer between GM1 (Cy5) and the different receptors was measured from the increase in donor (Cy3) fluorescence after acceptor (Cy5) photobleaching. The percentage of energy transfer and standard deviation was calculated from three independent experiments. Asterisks indicate statistically significant (*p*<0.05) increase in energy transfer compared to corresponding unstimulated controls. (B) Inhibition of IL-6 production after lipid raft disruption. Human amniotic epithelial cells were either not treated (white barcharts) or pre-treated with nystatin and subsequently stimulated with the different Ureaplasma serovars or MBA. The supernatants were harvested and assayed for cytokine content using the Cytometric Bead Array (CBA) system (Becton Dickinson). Fluorescence was detected using a FACSCalibur (BectonDickinson). The data represents the mean ± SD of three independent experiments. Asterisks indicate statistical significance (*p*<0.05).

It has been previously shown that regions of the plasma membrane known as lipid rafts, or microdomains facilitate bacterial LPS-induced cell activation [18]; [19]. In addition, we have demonstrated that TLR2 is also recruited within lipid rafts upon stimulation by its ligands [20]. Since we demonstrated that *Ureaplasma*-induced cellular activation is mediated mainly through TLR2 on the cell surface, we proceeded to determine whether TLR2 was recruited within lipid rafts upon stimulation by different *Ureaplasma* serovars or MBA. FRET experiments between TLR2 and GM-1 ganglioside were performed before and after stimulation by *Ureaplasma* serovars or MBA. TLR2 molecules were labelled with Cy3-TLR2 and GM-1 ganglioside, a raft-associated lipid, was labelled with Cy5-cholera toxin. It was shown that similarly to TLR2 ligands, *Ureaplasma* and MBA stimulation could recruit TLR2 in lipid rafts (Figure 5A).

Interestingly, TLR1 was found not to associate with GM1 ganglioside in response to *Ureaplasma* stimulation, thus suggesting that TLR1 is not involved in the innate immune recognition of *Ureaplasma*, whereas TLR6 was found to associate, leading us to believe that TLR2 heterodimerises with TLR6 in order to recognise *Ureaplasma* serovars.

In addition, although TLR4 has been shown to be recruited within these microdomains after enterobacterial LPS stimulation and this clustering is crucial for LPS-induced cytokine production [18], in this study it was shown that there was minimal recruitment of TLR4 within lipid raft compared to TLR2 in response to *Ureaplasma* stimulation.

In order to test the significance of TLR-lipid raft recruitment in response to *Ureaplasma*, we proceeded to disrupt lipid raft formation using a lipid raft-disrupting drug, such as nystatin. It was shown that when human amniotic epithelial cells were pre-treated with nystatin (leading to lipid raft disruption), IL-6 responses were inhibited (Figure 5B), suggesting that accumulation of TLR2 and TLR6 within lipid rafts in response to *Ureaplasma* is crucial for signalling and pro-inflammatory cytokine responses.

### Ureaplasma recognition intracellularly

**Figure 6:**
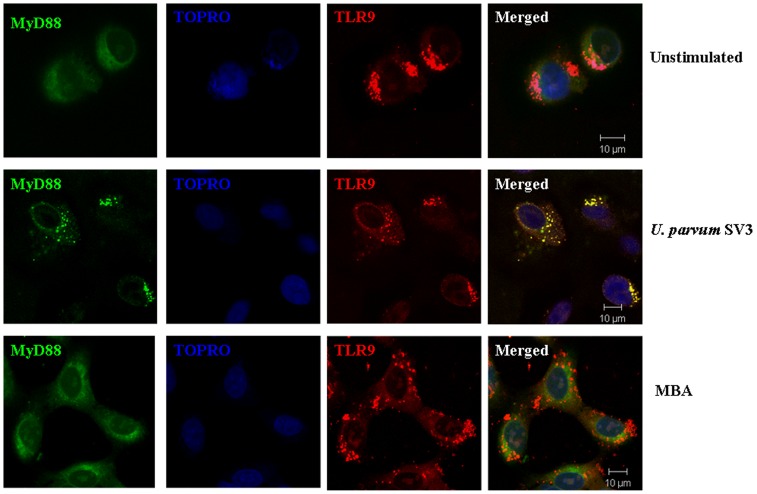
*Ureaplasma* internalization recruits MyD88 in endosomes. Human amniotic epithelial cells were either not stimulated (top panels) or stimulated with either *Ureaplasma* SV3 (middle panels) or MBA (bottom panels) and imaged using a Zeiss 510 META confocal microscope. Intracellular MyD88 was stained using a polyclonal antibody directly labelled with FITC. TLR9 were labelled using anti-TLR9-TRITC. TOPRO was used to label the nucleus of the cells. Merged images showing extensive overlay of areas positive for MyD88 and TLR9 are seen as yellow (Scale Bar, 10 µm).

In order to elucidate the intracellular interactions of *Ureaplasma* with TLR9 we employed confocal microscopy. Amniotic epithelial cells were stimulated with either *Ureaplasma* SV3 or MBA and in order to determine whether signal transduction occurs through TLR9 in the endosomes, we investigated the presence of MyD88, which is an obligatory adaptor protein for TLR signal transduction by using anti MyD88-TRITC. Our results showed that MyD88 is recruited in endosomes (Figure 6), where it colocalises with TLR9 (Figure 6, middle row), when the cells are stimulated with *Ureaplasma*. As expected, MBA stimulation did not recruit MyD88 to the endosome (Figure 6, bottom row), thus demonstrating that MBA is recognised on the cell surface, but once *Ureaplasma* internalizes its DNA triggers TLR9 recognition intracellularly.

## Discussion

Intrauterine infection is a common cause of preterm delivery. Chorioamniotitis or inflammation in the fetal membranes is associated with over 60% of preterm deliveries [21]. The most common organisms isolated from the amniotic fluid of women with chorioamnionitis are the *Ureaplasma* species [22]. These organisms are commonly found within the uterus in association with spontaneous preterm labour and with preterm PROM. In addition, the intrauterine presence of these organisms has been linked with an increased production of a wide variety of cytokines, matrix metalloproteinases and postaglandins, all believed to be in the causal pathway for PROM, thus activation of the innate immune system has been suggested [8]; [23].

*Ureaplasmas* are unusual bacteria in that they have a plasma membrane but lack a peptidoglycan cell wall, use urea as the sole source of energy, and are dependent on the host for other metabolic functions [24]. Since they lack a conventional bacterial cell wall, the question that remains is how they trigger the innate immune system and which PRRs they engage. It is imperative to elucidate how *Ureaplasma* species can induce an inflammatory response, if we are to understand how these bacteria cause PROM.

Although much attention has been paid to TLR expression and function in the female genital track [25]; [26], little is known about TLRs in the human amniotic epithelial cells and how they recognise *Ureaplasma* species. In this study, we set out to identify which TLRs are involved in triggering pro-inflammatory responses in response to *Ureaplasma* species in human amniotic epithelial cells. Initially we investigated whether *Ureaplasma* can trigger the secretion of pro-inflammatory cytokines in human amniotic epithelial cells. Our experiments suggest that *Ureaplasma SV2, SV3* and *SV14* are capable of inducing the secretion of pro-inflammatory cytokines, which include TNF-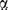
IL-6, IL-8 and IL-1β within the first two hours of their interaction with the host. No significant difference was observed among the different *Ureaplasma* serovars. Thus we proceeded to elucidate the mechanism by which *Ureaplasma* can induce a cytokine response in human amniotic epithelial cells. Since TLRs are the key receptors for sensing bacteria, we investigated whether TLRs were involved in the cytokine production in response to *Ureaplasma*. In order to examine which if any, of the TLR molecules are involved, we utilised HEK293 cells transfected with different TLRs. It was shown that *Ureaplasma* species were able to activate only cells transfected with either TLR2, TLR2/6 or TLR9 and to a lesser extent TLR4. This is partly in agreement with Shimizu et al. [9] where they suggested that TLR1, TLR2 and TLR6 are involved in sensing *Ureaplasma parvum*. In our study, we did not find any TLR1 involvement, whereas we found TLR9 involvement. Differences in the two studies might lie in the fact that the Shimizu et al study was performed with bacteria cultured in media supplemented with yeast extract, which itself constitutes a PAMP, thus results obtained might have been affected by the presence of yeast components.

Furthermore, similarly with the study by Shimizu et al. [9] we proceeded to investigate whether lipoproteins were triggering the response observed. It was shown that MBA was triggering responses via TLR2/6, thus suggesting that this heterodimer might be engaged on the cell surface prior to *Ureaplasma* internalization.

The ability to induce an inflammatory response via TLR9 seems to suggest that *Ureaplasma* species must internalise and replicate within the host's cells. Thus suggesting that the innate recognition of *Ureaplasma* in the early stages of attachment to the host cells is via the recognition of lipoproteins by TLR2/6, but once the bacteria have infected the cells TLR9 is the main initiator of the inflammatory response. This seems to be consistent with the fact that TLR9 is only expressed intracellularly, and thus the bacteria must internalise in order to interact with these receptors.

Interestingly, when we investigated whether *Ureaplasma* species could induce the formation of receptor clusters on the cell surface of human amniotic epithelial cells, it was shown that they could induce receptor clusters comprising of TLR2/6 on the cell surface. These clusters seem to form within lipid rafts. Since these pathogens trigger responses via TLR2, it is possible that CD36 could act as a key molecule within the receptor cluster. CD36 has recently been shown to associate with TLR2 [27] and this interaction might exacerbate the inflammatory response.

Furthermore, when we investigated whether TLR9 was triggering signalling from the endosomes in response to *Ureaplasma* infection, it was shown that following *Ureaplasma* infection, TLR9 co-localised in the endosomes with the signalling adaptor molecule MyD88. Therefore, our data suggests that *Ureaplasma* CpGDNA must be targeted to endosomal compartments upon internalisation. TLR9 is perfectly placed in endosomal compartments in order to be able to “sample” this endocytosed material and trigger cytokine response whenever this molecular “signature” is recognised.

Overall our data suggests that the inflammatory response triggered by *Ureaplasma* in amniotic epithelial cells is mediated by the synergic activation of multiple TLRs. It seems that a part of the inflammatory response is triggered by lipoproteins, such as MBA, in the initial stages of the bacterial attachment to the cell surface and is mediated via TLR2. MBA is a surface exposed lipoprotein, which can undergo size and phase variation *in vitro* and *in vivo*. It has been previously shown that MBA size variation is associated with the severity of chorioamnionitis in a pregnant sheep model of intra-amniotic *Ureaplasma* infection [28], therefore MBA size variation might influence the interaction of MBA with TLR2/TLR6 heterodimers and thus determine the intensity of the innate immune response and consequently the severity of amniotic fluid infection. One effective microbial strategy for avoiding host recognition is the modification/variation of PAMPs. Since our study demonstrates that MBA is a PAMP, the primary function of this antigenic variation could be to evade the innate immune response, and thus MBA variation could influence the virulence of the different strains. This might explain why certain *Ureaplasma* isolates are more associated with severe disease than others.

Although, it has been previously suggested that in addition to TLR2, TLR4 might be activated in response to *Ureaplasma* [29], in our study we only observed TLR2 activation in response to all *Ureaplasma* serovars tested. The fact that MBA engages TLR2, and not TLR4, might shed more light into the reasons why these organisms can develop chronic, low-level inflammation of amniotic epithelial cells leading to PROM. Activation via TLR2 results in a subdued inflammatory response [30] allowing the organism to establish a chronic foothold in amniotic epithelial cells.

Synergic inflammatory response of TLR2, TLR6 and TLR9 seem to produce a chronic inflammatory response against *Ureaplasma*, which could eventually lead to irreversible injury of fetal membranes. There are several clinical implications of our findings, since the current study is the first study into the mechanisms by which *Ureaplasma* species cause chronic inflammation of the amniotic epithelium, and might help us find new TLR-based therapeutic targets for *Ureaplasma*-induced chorioamniotitis.
