# Correction: Synergic Activation of Toll-Like Receptor (TLR) 2/6 and 9 in Response to *Ureaplasma parvum* & *urealyticum* in Human Amniotic Epithelial Cells

**DOI:** 10.1371/annotation/1dc00176-e096-4621-9494-2d848dac8262

**Published:** 2013-08-21

**Authors:** Martha Triantafilou, Benjamin De Glanville, Ali F. Aboklaish, O. Brad Spiller, Sailesh Kotecha, Kathy Triantafilou

A funding organization was incorrectly omitted from the Funding Statement. The first sentence of the funding statement should read: "This work was supported by a grant awarded from the Children & Young People's Research Network (NISCHR, Wales) as well as the Sir Ewen Maclean Bequest Fund in the Cardiff University School of Medicine." 

